# Sleep and Obesity Risk in Children: A Systematic Review of Multiple Dimensions of Sleep

**DOI:** 10.3390/children12121577

**Published:** 2025-11-21

**Authors:** Surendra Gupta, Purushottam Lal

**Affiliations:** 1 American Board of Pediatrics, Clinica Sierra Vista, Fresno, CA 93706, USA; 2 Pediatric Pulmonology, Yale New Haven Children Hospital, New Haven, CT 06511, USA; purushottam.lal@yale.edu

**Keywords:** pediatric population, obesity, body mass index, sleep, sleep dimensions

## Abstract

**Objective**: Our objective was to determine the relationship between sleep dimensions and obesity risk in the pediatric population. **Methods**: This systematic literature review was performed following PRISMA guidelines. Peer-reviewed original research focusing on sleep parameters in the pediatric population aged 0–18 years, with obesity risk measured using objective measures, was included. **Results**: The review analyzed 27 studies, comprising 85,669 participants aged 0–18 years. The majority of the studies observed an inverse relationship between short sleep duration and obesity risk, with insufficient sleep translating to negative weight status, increased waist circumference, and higher obesity prevalence. Sleeping late on both weekdays and weekends increases the risk of obesity. Only four studies assessed sleep quality, with inconsistent findings. Sleep duration and timing had a stronger effect on obesity risk than sleep quality. **Conclusions**: Sleep duration was an independent determinant of obesity risk in the pediatric population, while sleep timing and quality showed an inconsistent association.

## 1. Introduction

The rise in obesity among the pediatric population is a major global problem, as between 2000 and 2023, 8.5% of the total individuals under 18 years were categorized as obese [1]. Having a higher body mass index (BMI) and being obese during childhood increases the risk of cardiac conditions and diabetes, resulting in greater morbidity and mortality later in life. Risk factors for pediatric obesity range from genetic origin to environmental and lifestyle factors [2]. Modern lifestyles have led to higher screen time in children, negatively impacting sleep duration and sleep quality [3]. Studies from both developed and developing nations have revealed the detrimental effects of insufficient sleep on the physical, psychological, and social well-being of children [4,5].

The pediatric population who sleeps for a shorter time exhibits a higher likelihood of having an overweight or obese status than those with sufficient sleep [6]. A recent meta-analysis supported the hypothesis that children of the age group 6 to 10 years, who had fewer sleeping hours, had an elevated risk of getting obese [7]. Jarrin et al. carried out an investigation involving 240 Canadian children aged between 8–17 [8]. The study observed that the participants with late bedtimes and wake-up times had a statistically significant elevated risk of negative weight status, in contrast to those reported with earlier sleep schedules. Similarly, research conducted in the Australian pediatric population demonstrated that sleeping late was an independent determinant of high BMI z-scores and poor food intake quality scores [9].

Inadequate sleep causes hormonal alterations that affect weight and nutritional intake [10]. Additionally, restricted sleep may impact metabolism and have a thermic effect on food [10]. Shorter sleep duration disrupts carbohydrate metabolism and leads to glucose intolerance, further leading to a heightened risk of obesity. Insufficient sleep may also reduce body energy levels throughout the day, reducing physical activity and energy expenditure, indirectly contributing to increased weight [11,12]. Therefore, potential underlying mechanisms such as metabolic dysregulation, reduced energy levels, and impaired weight regulation resulting from insufficient sleep cumulatively add up to a negative weight status in the pediatric population.

Apart from sleep duration, other dimensions like sleep timing and sleep quality are being extensively investigated for their potential effect on obesity in adults [13]. However, there is a lack of sufficient literature informing on the role of these two dimensions of sleep (timing and quality) and obesity risk in the pediatric population. Children experiencing poor sleep quality may present with higher BMI and be predisposed to developing obesity in the future [14]. Thus, sleep timing and sleep quality potentially serve as independent determinants of obesity risk.

Given the increasing number of investigations determining the multi-dimensional effect of sleep on obesity across different pediatric age groups, a systematic analysis is warranted. Previous investigations have predominantly analyzed the effect of sleep on obesity using sleep duration as the sole parameter, overlooking other key sleep dimensions [3,15]. Furthermore, although some studies have examined multiple dimensions of sleep, their study populations have been limited to either children or adolescents rather than encompassing the entire young population in the age group 0–18 years [16,17]. Consequently, the broader impact of various sleep parameters on obesity risk across different developmental stages remains inadequately understood.

To address this, the present systematic literature review is aimed to assess the relationship between sleep characteristics (namely duration, quality and timing) and risk of overweight and obesity for the pediatric age groups, 0–18 years. More specifically, this review examined if shorter or longer sleep duration was related to obesity risk in infants, children, and adolescents; if or how sleep timing factors (for example, late bedtimes and irregular schedules) impact obesity-related outcomes; and the effect of sleep quality (like night waking and poor regulation) on obesity risk. Furthermore, we compared results between pediatric age groups and study designs to provide the clearest picture of how different aspects of sleep are associated with weight status across growth and development.

## 2. Materials and Methods

### 2.1. Search Questions

The research question “In a pediatric population aged 0–18 years what is the association between sleep parameters (duration, quality, timing), on the risk of overweight and obesity?” was structured using PECO (Population, Exposure, Comparison, Outcome) (Table 1).

### 2.2. Literature Search

The Preferred Reporting Items for Systematic Reviews (PRISMA) guidelines were followed throughout the conduct of the present systematic literature review. The electronic databases, including PubMed, Google Scholar, and Cochrane, were systematically searched for publications from 2015 to 2025. A search strategy based on unique keywords and MeSH terms was used to include all possible sleep dimensions and additional terms that could demonstrate the obesity risk in the age group 0–18 years. The search strategy combined controlled vocabulary (MeSH) and free-text terms: (“Sleep” [MeSH] OR “Sleep Quality” OR “Sleep Duration” OR “Sleep Timing”) AND (“Obesity” [MeSH] OR “Overweight” [MeSH] OR “Body Mass Index” OR “Waist Circumference”) AND (“Child” [MeSH] OR “Adolescent” [MeSH] OR “Infant” [MeSH]). Boolean operators “AND” and “OR” were used to combine terms appropriately across PubMed, Cochrane Library, and Google Scholar. The final literature search is carried out on 30 March 2025.

### 2.3. Eligibility Criteria

Studies were included for analysis after screening based on a pre-defined selection criteria, that included: (a) original research papers, peer-reviewed; (b) study participants included infants, children, or adolescents (0–18 years of age); (c) assessment of sleep duration and sleep and sleep timing as a primary objective; (d) studies that incorporated study design, such as cohort, cross-sectional, case–control, or observational; (e) association between sleep duration and risk of obesity: (f) obesity risk measured by BMI, waist circumference, or body fat percentage; (g) studies published in English language from 1 January 2015–1 January 2025.

Studies were excluded if (a) study participants aged >18 years; (b) reviews, abstracts, unpublished studies, and editorials; (c) they did not assess the role of sleep duration on the risk of obesity; (d) studies that included several confounding factors that influenced the direct impact of sleep on the risk of obesity among the pediatric population.

### 2.4. Data Extraction

Two reviewers (GC and FB) independently performed data extraction and assessed the quality of the studies. The study details extracted from the articles included information about the author, study design, number of participants, nation of origin, age group of the study sample, assessment of sleep quality, and obesity (odds ratios or relative risks of negative weight status). The dimensions of sleep were categorized as sleep quality, timing (wake time and bedtime), and duration. Obesity risk was predominantly measured using BMI, waist circumference, and body fat percentage. A summary of the findings was added for each study. Sleep parameters were assessed using validated questionnaires or objective tools across studies. Parent-reported sleep measures most frequently used the Children’s Sleep Habits Questionnaire (CSHQ), Brief Infant Sleep Questionnaire (BISQ), or structured sleep diaries, while self-reported data in adolescents commonly employed the Pittsburgh Sleep Quality Index (PSQI) or Youth Risk Behavior Survey (YRBS) sleep items. Objective measurements were obtained via actigraphy or accelerometry-based devices, which provide continuous monitoring of sleep–wake patterns. When studies did not explicitly mention an instrument, the assessment method was classified as “general self/parent report.” These categorizations were used to ensure comparability of sleep assessment methods across the included studies.

### 2.5. Study Quality

The quality of all the studies was determined using the modified Newcastle-Ottawa Scale (NOS) [18]. The NOS ranges from zero to ten, with higher scores representing better study quality. The tool assesses cohort study quality across three domains. Selection of the study participants (five stars) evaluates cohort definition, comparability of the study participants (two stars) assesses confounder control, and outcome of the study (three stars) examines measurement and follow-up. This star-based system ensures standardized study evaluation. Studies scoring seven to ten stars are categorized as high-quality, four to six stars as moderate quality, and between zero and three stars are classified as low quality.

## 3. Results

### 3.1. Search Result

**Figure 1:**
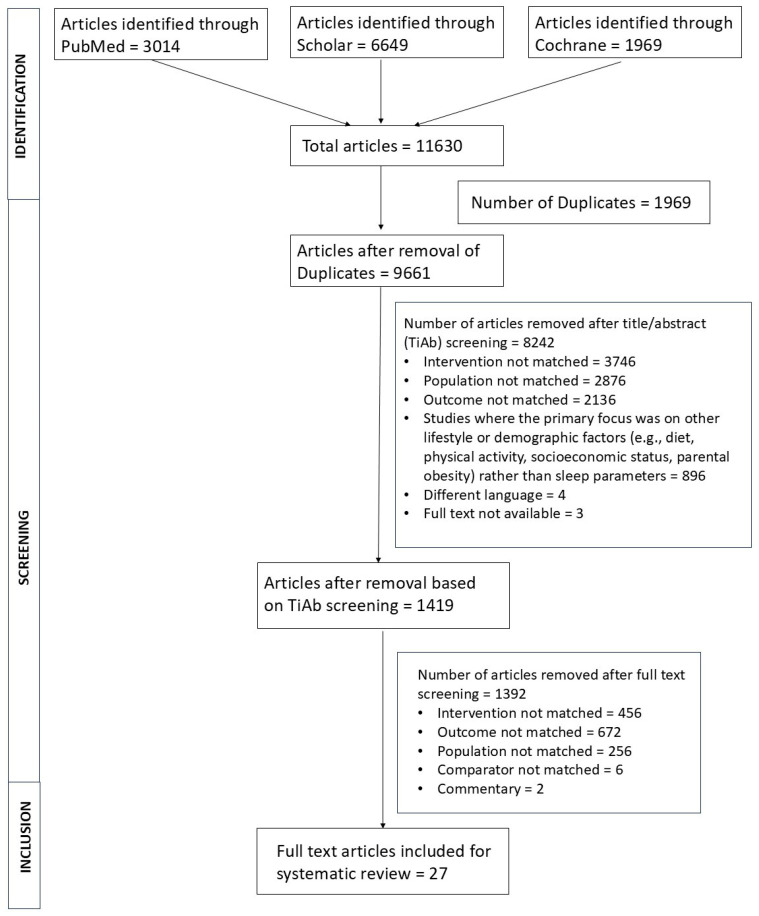
PRISMA flow diagram.

Multiple literature databases, including PubMed, Google Scholar, and Cochrane, were used for the initial identification of studies using a tailored search strategy. The database search resulted in the identification of 11,630 articles. Following duplicate removal, 9661 studies remained, out of which 8242 studies were irrelevant and excluded, resulting in a screening of 1419 studies. Of these, 1392 studies were excluded (456 for intervention mismatch; 672 for irrelevant outcomes; 256 for population mismatch; 6 that did not have a relevant comparator; and two articles that were commentaries). This left 27 articles quantitatively assessing the relation between obesity and different sleep dimensions for inclusion within the current study (Figure 1). The overall quality of the studies ranged between 3 and 9 on the quality assessment scale. A major percentage of included investigations scored either 6 or 7, indicating that most were of moderate to high quality.

### 3.2. Study Characteristics

Individual study characteristics are outlined in Table 2. From a total of 27 studies, 85,669 participants were included for analysis [19,20,21,22,23,24,25,26,27,28,29,30,31,32,33,34,35,36,37,38,39,40,41,42,43,44,45]. The studies assessed the entire pediatric population ranging from 0 to 18 years, with certain longitudinal studies following participants from infancy into childhood. A significant percentage of studies were cross-sectional (15 studies) [20,21,22,23,24,26,28,31,32,36,37,38,39,44,45], while 7 studies were longitudinal [19,27,30,33,34,35,43], and 4 studies employed both longitudinal and cross-sectional study designs [25,29,40,41]. A single study was “a secondary analysis of a previously published randomized controlled trial (RCT)” [42]. The geographical distribution of study participants varied, with research conducted in North America (USA, Canada), Europe (UK, Netherlands, Sweden, Italy), Asia (China, Korea, Kuwait), South America (Chile, Brazil), and Oceania (New Zealand, Australia). Across the 27 included studies, 13 used parent-reported sleep measures (mostly based on the CSHQ or BISQ), 9 used self-reported questionnaires such as the PSQI or YRBS, and 4 used actigraphy-based objective measures. One study combined both accelerometry and parental reporting. This classification clarifies the assessment methodology beyond the simple self/parent distinction.

Table 3 details the outcome of various sleep dimensions on obesity measures. Sleep parameters were assessed using different methods. The most commonly used technique was parent-reported sleep measures, used in 13 studies [19,22,25,26,27,28,30,31,33,35,41,43,45], followed by self-reported sleep dimension in 9 studies [20,21,24,34,36,37,38,39,44]. Objective sleep measurements using accelerometry were used in 4 studies [23,29,32,40], and one study used a combination of parent reports and accelerometry for sleep assessment [42]. All studies except one [34] used BMI to classify children into various weight statuses, based on established criteria outlined by the World Health Organization (WHO), Centers for Disease Control and Prevention (CDC), International Obesity Task Force (IOTF), or China. Apart from BMI, waist circumference was used as an additional measure in 4 studies [23,34,39,44], while body fat percentage was reported in 2 studies [23,39].

### 3.3. Sleep Duration

A large number of studies included within the review support a negative association between sleep duration and obesity risk. This indicates that a shorter sleep duration significantly increases the likelihood of an overweight or obese weight status in the pediatric population [19,20,22,23,24,25,26,27,31,32,34,35,38,39,44,45]. Several studies demonstrated that the pediatric population receiving <10 h of sleep at night was predisposed to having a higher BMI, increased waist circumference, and a greater risk of obesity [28,33]. Furthermore, studies also highlighted that insufficient sleep during early childhood is a predictive factor for obesity in later years [27,35]. Larsen et al. (2017) identified a gender-specific obesity risk, with shorter sleep durations having a significant influence on the BMI of boys but not among girls [45]. Despite the strong association between inadequate sleep and the risk of obesity in young individuals, few studies have reported contrasting results. Seven studies found no direct association between the risk of obesity with shorter sleep durations [21,29,30,40,41,42,43].

Several longitudinal investigations have studied the temporal relationship between sleep duration and changes in BMI, yielding mixed results. Alamian et al. (2016) observed a direct correlation between infant sleep problems with an elevated risk of high BMI during later childhood [27]. In contrast, Derks et al. (2019) identified an insignificant association between mid-childhood sleep duration and body composition at mid-adolescence [43].

Six studies, including infants, reported no significant relationship between sleep duration during infant years and high BMI later in childhood [30,40,41,43]. However, only two studies found that less sleep time in infants can be a significant predictor of obesity in the future [31,35]. Bonuck et al. (2015) and Wang et al. (2016) reported that short sleep duration in toddlers (ages 4–7 years) significantly elevated the likelihood of an obese weight status after a few years [19,33]. Therefore, the findings highlight that the impact of shorter sleep duration and obesity risk may potentially depend on the age group, with a more pronounced effect on toddlers than infants.

### 3.4. Sleep Timing

Out of 27 studies, only 6 studies investigated the effect of sleep timing on children’s BMI [21,23,25,31,34,38]. Children going to bed late on both weekdays and weekends were at risk of overweight and obese status [21]. Similarly, Scharf et al. (2015) and Alqaderi et al. (2017) found that late bed times led to significantly increased risk of obesity and higher waist circumference among the pediatric population [25,34]. Ekstedt (2016) reported a direct correlation between late bed time and higher BMI [31]. Conversely, McNeil et al. (2015) and Rosi et al. (2017) observed an insignificant impact of sleep timing on BMI in children [23,38]. The lack of consistent results across the studies limits the direct link between late bedtimes and sleep times and obesity risk.

### 3.5. Sleep Quality

Only four studies measured sleep quality among the participants [27,32,36,42]. Only Martoni et al. (2016) assessed sleep quality based on the amplitude of circadian rhythm, identifying a significant correlation between low amplitude and high BMI [32]. Another study observed sleep disturbances as a risk for being overweight, while their effect on obesity was statistically insignificant [27]. García-Hermoso et al. (2017) [36] and Zhang et al. (2019) [42] found an insignificant correlation between poor sleep quality and obesity risk in young individuals. This suggests that sleep duration and timing tend to have a more critical role than overall sleep quality [36,42].

## 4. Discussion

The present systematic literature review aimed at evaluating the effect of improper sleep patterns using sleep duration, quality, and timing on weight status in the pediatric population. The majority of the studies observed an inverse relationship between daily hours of sleep and obesity measures, with higher sleep durations translating to significantly lower BMI, waist circumference, and body fat percentages. However, a few contradictory studies found no such association. While duration of sleep was identified as the strongest predictor of obesity risk, sleep timing also showed a moderate effect on body composition, with later bedtimes contributing to a higher risk of being overweight and obese. In contrast, sleep quality did not demonstrate a significant impact on obesity outcomes. Nonetheless, including these non-conventional parameters of sleep in the review adds significant value. The overall findings support the notion that sleep is an essential factor for regulating health and preventing obesity in the pediatric population.

There are several biological phenomena modulated by sleep, likely the reason behind the strong relation between sleep and obesity. Although the precise hormonal and molecular pathways are not well-studied, impaired sleep results in metabolic dysfunction and energy imbalances. Sleep deprivation can trigger sympathetic nervous system activation, which increases levels of lipid-modulating compounds in the body, such as catecholamine and cortisol [3]. Furthermore, an elevation of inflammatory markers—interleukins and tumor necrosis factor-alpha (TNF-α)—is observed [46]. Growth hormone and leptin concentrations diminish in those with short sleep durations, both of which are involved in energy metabolism [3]. Leptin deficiency reduces satiety, increasing appetite and weight gain [10,47]. Growth hormone stimulates lipolysis, and its suppression due to inadequate sleep may contribute to increased fat mass [48]. Therefore, sleep deprivation can lead to significant alterations in metabolic concentration, promoting obesity.

Inadequate sleep may also translate to a negative weight status due to disrupted energy homeostasis. Short sleep duration increases energy intake and reduces energy expenditure, causing unwanted caloric intake which was otherwise not required [49]. Sleep restrictions potentially induce fatigue, which lowers the motivation for engaging in physical activity and spontaneous movement, further exacerbating weight gain [50]. Melatonin regulates involuntary energy expenditure, which is suppressed due to late-night light exposure. This decreases energy expenditure while accumulating fat in the adipose tissues [51]. The multiple metabolic and regulatory pathways disturbed due to a lack of sleep demonstrate the importance of sufficient sleep during childhood to prevent pediatric obesity.

A significant proportion of the included literature focused on sleep duration in children. Several previous systematic reviews and meta-analyses have investigated the role of duration of sleep in regulating BMI [3,15]. The included studies predominantly report an inverse relationship between sleep duration and obesity risk, indicating that short sleep durations may be an underlying factor in the obese or overweight pediatric population. This finding is supported by a large body of evidence [52,53,54]. Li et al. (2017) conducted a meta-analysis, reporting a pooled risk ratio of 1.3 between short sleep duration and obesity [55]. Similarly, in another meta-analysis, the adjusted risk ratio for obesity was observed to be 1.57 and 0.83 in short sleep duration and long sleep duration, respectively. This represents a 57% higher risk of obesity in the pediatric population sleeping for less time, while 17% lower risk in those receiving long sleep times [3]. Fatima et al. (2015) systematically found that individuals with shorter sleep durations nearly doubled the odds of developing excess adiposity compared to their counterparts who got adequate sleep [52]. Albeit the differences in study type, population, and measures, the cumulative evidence positions short sleep durations as a strong determinant of weight status in children.

A novel finding of the study is the differential effect of less sleep among different age groups. Inadequate sleep was found to have a stronger risk of obesity in toddlers and adolescents, while infants showed a moderate effect. Miller et al. (2018) reported contrasting results, with a consistent risk of obesity observed across all age groups [15]. This can be attributed to the unequal distribution of participants within different age categories in the present study. It is hypothesized that infant sleep patterns may be more flexible and reversible, with insufficient sleep during this period not necessarily leading to long-term weight gain. However, as children grow older, shorter sleep durations become a stronger predictor of obesity, indicating that sleep habits established in toddlerhood may have a lasting impact on metabolic health. Further investigation is required to confirm the hypothesis.

While sleep duration emerged as an independent determinant of childhood obesity, other dimensions of sleep, such as quality and timing, show inconsistent associations with weight status. The risk of high BMI due to late bedtime was reported in several studies. Similar to the present results, Morrissey et al. (2020) showed that the pediatric population with late bedtimes had a greater risk of being obese, while such an association for late wake times was not reported [16]. This association between late bedtime and obesity can be due to its effect on sleep duration. As the study population comprises the pediatric population, likely school-going, they tend to have a consistent wake time. Conversely, bedtimes are subject to individual variability [56]. This may make late bedtime the cause of shorter sleep duration, as the wake time remains relatively uniform for a child. Therefore, the positive association between bedtime and BMI can be attributed to the reduced hours of sleep in the pediatric population.

Another potential explanation is that children who sleep late at night may adopt poor behavioral habits like increasing screen time and unhealthy snacking. Busto-Zapico et al. (2014) observed that children with frequently late bedtimes mostly spent the additional hours engaging in sedentary activities like video games and television [57]. Thivel et al. (2015) indicated an increased consumption of snacks and sugary beverages in the pediatric population with late bedtimes [58]. Therefore, late bedtimes may not only reduce sleep duration and disrupt energy homeostasis, but also develop negative behaviors like sedentary activities and high caloric consumption, which are known factors for obesity.

The studies either used sleep midpoint or sleep–wake patterns to measure sleep timing. However, the reliability of these parameters in sleep timing analysis is in question [16]. Furthermore, the low number of studies informing on this parameter further limits the ability to draw conclusive results. The causal relationship between late bedtime or wake time with obesity cannot be determined due to the absence of any longitudinal studies investigating these parameters. Further investigations using objective parameters of sleep timings are required to strengthen their link with obesity.

Sleep duration is sometimes considered a proxy for sleep quality. However, these two are distinct sleep dimensions, with sleep quality having a profound impact on emotional, behavioral, and cognitive function [59]. To confirm the true effect of inadequate sleep on obesity, it is recommended to consider both sleep duration and sleep quality in tandem. Despite its significance, the present study identified only a few studies evaluating obesity risk with poor sleep quality, with no definite correlation between the two variables. This is in alignment with the results of a systematic review on school-aged children [16]. Their analysis revealed that over half of the sleep quality studies reported no significant association, similar to the present findings. Further high-quality studies are required to confirm whether poor sleep quality can result in negative weight status in the pediatric population.

## 5. Study Implications

The present study comprehensively examines multiple sleep dimensions and their effect on obesity across different age groups.

A shorter sleep duration in infancy has often been associated with a higher body mass index (BMI) level or increased risk of overweight in childhood (e.g., Derks 2017 [35]; Alamián 2016 [27]), but other studies including those by Taylor (2018) [40], Wang (2019) [41], and Derks (2019) [43] did not find consistent or long-term predictive relations between infant sleep duration and later BMI. Qualitative aspects of sleep also seem to factor in, with infant sleep problems like napping and the inability to have good sleep regulation reported as predictors of overweight but not necessarily obesity (Alamián 2016). There have been reports of higher BMI in toddlers with later bed times and shorter total sleep time (Ekstedt 2016 [31]). However, relationships between short sleep duration and weight problems or overweight in toddlers are generally weak or non-significant (Halal 2016 [30]). Within children sleep duration has been consistently associated with greater obesity and overweight risk (Cao 2015 [20]; Wang 2016 [33]; Agüero 2016 [26]; Anujuo 2016 [28]; Khan 2015 [21]), while more limited studies suggest bed time is associated with obesity risk (Khan 2015; Scharf 2015 [25]; Alqaderi 2017 [34]). There are mixed findings regarding sleep quality, with some papers showing valid associations (Martoni 2016 [32]; McNeil 2015 [23]) and others showing no meaningful relationship (García-Hermoso 2017 [36]). Importantly, effect sizes are large in this research area, with children sleeping <7–9 h showed an increased risk of obesity of >30–60%, while the limited amount of sleep in adolescents has been shown to increase obesity, overweight, and abdominal obesity risk (Seo 2019 [44]). There is a defined risk gradient where the greatest risk was seen in very short sleep duration (≤5–6 h). There appeared to be little to no additional protective effect to sleep duration by simply sleeping longer than the optimal time. Duration of sleep has importance across the lifespan, but the strongest and most consistent associations with obesity appear in school-age children and adolescents, where findings in infancy and toddlerhood are much less consistent.

Apart from the commonly discussed parameter of sleep duration, this study assesses the use of sleep timing and quality as predictors of weight status. However, due to the smaller amount of included literature investigating these two dimensions, the evidence remains inconclusive. Nonetheless, it forms the basis for further high-quality research to investigate these parameters and define the underlying mechanism for their association. Given the increased screen time, late bed times, and changing parent routines, a social jetlag between the biological and social clock in the pediatric population is expected. The study underscores the detrimental effects of impaired sleep in the pediatric population on body composition. Therefore, the sync between the circadian cycle must be maintained, which will ensure optimal body metabolism and overall health.

Within the studies included for systematic analysis, there was inconsistency in the measures of sleep duration and their definition. This led to heterogeneous results, highlighting the need for uniform methodologies in future research. Furthermore, the study underscores the urgent need for standardized guidelines on optimal sleep duration and timing, which could aid in developing targeted interventions to mitigate obesity risk. The present study aims to inform public health strategies and clinical recommendations on sleep, for the timely prevention of obesity in the pediatric population, which has negative effects in the future if it remains uncontrolled.

## 6. Strengths and Limitations

This systematic review is the first to include the complete pediatric population aged 0 to 18 years while simultaneously investigating 3 dimensions of sleep–duration, timing, and quality– as per the authors’ knowledge. A key strength of this review is the inclusion of high-quality studies, which provides confidence in the reliability and validity of the conclusions drawn. Additionally, objective measures of obesity, such as BMI, waist circumference, and body fat percentage, were included to ensure an accurate quantitative representation of weight status.

Despite the significant strengths of this study, several limitations must be acknowledged. First, only a few studies investigated sleep quality and sleep timing compared to sleep duration, limiting the ability to make conclusive statements about their relation with obesity. Second, the review included relatively few longitudinal studies. Therefore, the study cannot establish a causality between sleep parameters and obesity. Furthermore, cross-sectional designs were mainly employed, which cannot rule out the possibility of reverse causality, where obesity might contribute to shorter sleep durations. Third, a meta-analysis was not feasible as the results had significant inconsistencies and heterogeneity in reporting. Furthermore, the broad age range (0–18 years) utilized in this review could introduce bias, as physiological sleep needs differ significantly between infants, children, and adolescents. While Google Scholar was used to support and broaden the search strategy, it has its shortcomings in systematic reviews. These include replicability limitations in searching, lack of unique identifiers, and inconsistency in fair indexing. So, although it supplemented the PubMed and Cochrane, it should be interpreted cautiously. Lastly, the definition of short and long sleep duration varied between studies, leading to heterogeneous results. Hence, the study strongly supports the association between sleep and obesity, while improvements in study designs and methodologies can provide better insights.

## 7. Conclusions

The goal of this systematic review was to estimate the association between sleep duration, timing, and quality with obesity risk in the pediatric population (0–18 years). Overall, the results show that sleep duration was related to obesity risk along the evidence spectrum during all the age periods of interest. Inadequate sleep duration resulted in children with shorter sleep durations having greater BMI, waist circumference, and higher body fat percentage than peers sleeping longer durations. In contrast, inadequate sleep timing, for example late bed times, and sleep quality presented with conflicted or inconsistent associations with obesity risk which suggests more high-quality research on sleep timing, and sleep quality is warranted. Perhaps most notable, the effects of short sleep were stronger in toddlers and adolescents than in infants, illustrating that certain age periods may be more vulnerable than others. Most of the included studies examined were cross-sectional and therefore limited any causal inference about the relationship between sleep and obesity risk, the longitudinal data presented mixed conclusions about the sleep and obesity association which highlights the importance of the study design for interpreting findings. Overall, the evidence from this systematic review adds that sleep duration is the most important indicator regarding pediatric obesity risk. More-well-defined measures and definitions of sleep timing and quality need to occur to establish a causal link and develop public health recommendations pertinent to specific ages.

## Figures and Tables

**Table 1:** Components of the PECO question used to define eligibility criteria and study parameters.

Abbreviation	Descriptor	Elements of Question
P	Population	Pediatric population aged 0–18 years (infants, children, and adolescents)
E	Exposure	Sleep parameters (duration, quality, and timing)
C	Comparison	Variations in sleep parameters (e.g., short vs. adequate sleep, poor vs. good quality, early vs. late timing)
O	Outcome	Risk of obesity and overweight, assessed using body composition parameters and international references for weight status classification (BMI, waist circumference, adiposity indices)

**Table 2:** Population and study characteristics.

First Author (Year)	Study Design	Sample Size	Country	Age (Years)	Measure of Sleep	Measure of Obesity	Quality of the Study
Bonuck (2015) [19]	LGS	1899	United Kingdom	15	PR	BMI with IOTF criteria	7
Cao (2015) [20]	CSS	11,830	China	6–18	SR	BMI with Chinese criteria	6
Khan (2015) [21]	CSS	5560	Canada	10–11	SR	BMI	7
Labree (2015) [22]	CSS	1943	Netherlands	8–9	PR	BMI with IOTF criteria	6
McNeil (2015) [23]	CSS	567	Canada	9–11	ACC	Waist circumference, BMI with IOTF criteria, Body fat%	8
Peach (2015) [24]	CSS	1364	USA	11–13	SR	BMI	7
Scharf (2015) [25]	CSS, LGS	7000	USA	5 (from 4)	PR	BMI z-score with CDC criteria	4
Agüero (2016) [26]	CSS	1810	Chile	6–11	PR	BMI	8
Alamian (2016) [27]	LGS	895	USA	11 ± 2	PR	BMI z-score with CDC criteria	7
Anujuo (2016) [28]	CSS	2384	Netherlands	5	PR	BMI with IOTF criteria	6
Butte (2016) [29]	CSS, LGS	111	USA	3–5	ACC	BMI	7
Halal (2016) [30]	LGS	4231	Brazil	4 (from 1)	PR	BMI z-score with WHO criteria	6
Ekstedt (2016) [31]	CSS	167	Sweden	1	PR	BMI	5
Martoni (2016) [32]	CSS	115	Italy	10	ACC	BMI	5
Wang (2016) [33]	LGS	16,028	China	5 (from 3)	PR	BMI with Chinese criteria	6
Alqaderi (2017) [34]	LGS	6316	Kuwait	12 (from 10)	SR	Waist circumference	7
Derks (2017) [35]	LGS	5161	Netherlands	6 (from 2 months)	PR	BMI z-score	7
García-Hermoso (2017) [36]	CSS	395	Chile	12–13	SR	BMI with IOTF criteria	7
Del Pozo-Cruz (2017) [37]	CSS	1812	New Zealand	5–14	SR	BMI	6
Rosi (2017) [38]	CSS	690	Italy	9–11	SR	BMI z-score with WHO criteria	3
Wang (2017) [39]	CSS	5518	China	9–12	SR	BMI with Chinese criteria Body fat % Waist circumference	9
Taylor (2018) [40]	CSS, LGS	802	New Zealand	5 (from birth)	ACC	BMI z-score with WHO criteria	7
Wang (2019) [41]	CSS, LGS	2308	Netherlands	5 (from 0.5)	PR	BMI z-scoreghan with WHO criteria	6
Zhang (2019) [42]	Secondary analysis of an RCT	173	Australia	1–2.5	ACC, PR	BMI	8
Derks (2019) [43]	LGS	336	Australia	14 (from 2 months)	PR	BMI	6
Seo (2019) [44]	CSS	6048	Korea	10–18	SR	Waist circumference and BMI	7
Larsen (2017) [45]	CSS	206	Netherlands	7–12	PR	BMI z with CDC criteria	4

LGS: Longitudinal study; CSS: Cross-sectional study; PR: Parent reported; SR: Self-reported; ACC: Accelerometry; BMI: Body mass index; IOTF: International Obesity Task Force; CDC: Centre for Disease Control and prevention; USA: United States of America; WHO: World Health Organization. PR = Parent Report (e.g., Children’s Sleep Habits Questionnaire—CSHQ or Brief Infant Sleep Questionnaire—BISQ); SR = Self-Report (e.g., Pittsburgh Sleep Quality Index—PSQI, Youth Risk Behavior Survey—YRBS); ACC = Accelerometry/Actigraphy-based measurement.

**Table 3:** Study findings reporting the quantitative association between sleep dimensions and obesity/overweight risk.

First Author (Year)	Study Objective	Follow-Up Period	Sleep Duration	Sleep Timing	Sleep Quality	OB/OW Outcome	Study Summary
Bonuck (2015) [19]	To assess the correlation between the prevalence of obesity and sleep duration	15 Years	Sleep duration at 6.75 years Short Average Long			OR for OB (95% CI) 1.50 (1.00 to 2.25) 1 0.94 (0.58 to 1.54)	Short sleep duration during early life (approximately 7 years) is a significant risk factor for developing obesity later in childhood.
Cao (2015) [20]	To assess the correlation between the prevalence of obesity and sleep duration		Distribution of children based on sleep duration ≥9 h 7–9 h <7 h			OR for OB (95% CI) 1 1.328 (1.324–1.332) 1.581 (1.572–1.590)	Receiving fewer hours of sleep during the night significantly increased the risk of obesity, with those reporting sleep duration <7 h daily demonstrating a 58% higher chance of being obese.
Khan (2015) [21]	To determine the effect of varying sleep duration and timing on body weight status		Sleep duration (Hours per day)	Bed time on weekdays 9–10 p.m. After 10 p.m. Bed time on weekends 9–10 p.m. After 10 p.m.		OW: 0.86 OB: 0.75 OW: 1.33 OB: 1.48 OW: 1.62 OB: 2.64 OW: 1.2 OB: 1.43 OW: 1.6 OB: 1.26	No significant correlation was found between the number of hours of sleep per day and the risk of obesity or overweight status. However, the timing of the sleep schedule showed a significant impact. Late bed times on both weekdays and weekends increased the risk of being overweight, with the effect being particularly pronounced for obesity outcomes.
Labree (2015) [22]	To identify underlying lifestyle factors, including sleep duration, on negative weight status					Regression coefficient between sleep duration and BMI (95% CI) −0.12	An additional 30 min of sleep significantly correlated with a decrease in BMI by 0.24. Increased sleep duration slightly lowers the likelihood of being overweight or obese.
McNeil (2015) [23]	To assess the association between multiple sleep dimension and obesity measures					Regression coefficient with sleep parameters and waist circumference Sleep duration: −0.18 Sleep timing midpoint: 0.07 Bed time: - Wake time: −0.04 Regression coefficient with sleep parameters and BMI Sleep duration: −0.16 Sleep timing midpoint: 0.06 Bed time: −0.00 Wake time: −0.04 Regression coefficient with sleep parameters and Body fat% Sleep duration:−0.12 Sleep timing midpoint: 0.13 Bed time:−0.04 Wake time: −0.05	Sleep duration and the midpoint of sleep timing demonstrated a significant negative association with anthropometric outcomes.
Peach (2015) [24]	To assess the correlation between BMI and sleep duration among children					Correlation coefficient between BMI and sleep duration School night sleep duration: −0.15 Weekend sleep duration: −0.08	Higher duration of sleep during school nights and weekends demonstrated a significant correlation with lower BMI in children.
Scharf (2015) [25]	To identify any correlation between sleep timing and weight gain in children.	1 year		<1 standard deviation below mean Bed time > 1st deviation above mean Wake time <1 standard deviation below the mean		OR (95% CI) OW: 1.15 (0.93–1.42) OB: 1.48 (1.13–1.93) OR (95% CI) OW: 1.20 (1.04–1.39) OB: 1.26 (1.05–1.52) OR (95% CI) OW: 1.05 (0.86–1.28) OB: 1.20 (0.94–1.53)	Fewer hours spent sleeping was a significant modifiable factor for obesity. Late bed times were significantly correlated with the incidence of negative weight status among children.
Agüero (2016) [26]	To assess the correlation between insufficient sleep (weekdays and weekends) and children’s BMI		Percentage of children sleeping fewer than recommended hours Weekdays: 49.9% Weekends: 16.7%			OR for OB/OW (95% CI) 1.82 (1.29–2.56) 1.22 (0.95–1.57)	Children sleeping for fewer hours than the recommended guidelines reported a significantly higher risk of being overweight and obese. Children having short sleep duration during the weekdays had a greater risk compared to those with short night sleeps over the weekend
Alamian (2016) [27]	To determine the correlation between insufficient sleep during infancy with the risk of obesity during childhood.	13 years			Percentage of children presenting with sleep Problems according to different definitions Zuckerman:24.9% Richman: 12.6% Lozoff: 20.6%	OR (95% CI) OW = 1.62 (1.08–2.43) OB = 0.98 (0.65, 1.48) OW = 1.70 (1.03–2.78) OB = 0.79 (0.45–1.41) OW = 0.92 (0.58–1.45) OB = 0.77 (0.49–1.21)	A statistically significant positive association between infant sleep problems and the risk of being overweight during childhood was reported.
Anujuo (2016) [28]	To identify the impact of varying sleep duration with obesity among 5-year-old children		Percentage of children reporting short sleep durations of less than 10 h % Dutch: 11.3 Turkish: 38.0 Moroccan: 37.3 African-Surinamese: 37.6 Ghanaian: 53.1			Regression coefficient for short sleep and being OW (95% CI) Dutch: 1.61 (1.04–2.49) Turkish: 0.71 (0.33–1.56) Moroccan: 1.78 (1.01– 3.15) African-Surinamese: 1.86 (0.79–4.40) Ghanaian: 0.59 (0.20– 1.72)	Dutch and Moroccans reported a significant correlation between short sleep durations and overweight status.
Butte (2016) [29]	To identify the predictive potential of sleep duration for changes in BMI after 1 year.	1 year	Minutes per day			Regression coefficient for sleep duration and BMI −0.004	Sleep duration was not identified as a predictor of BMI after 1 year
Halal (2016) [30]	To assess the impact of less sleeping hours during infancy on weight at an early age	3 years				PRs (95% CI) for OW/OB when children receive less sleep 1.26 (0.98; 1.62)	No statistically significant difference in the prevalence of negative weight status among infants was reported due to short sleep durations.
Ekstedt (2016) [31]	To assess the impact of sleep parameters of the risk of obesity in infants aged 1 year					Pearson correlation coefficient between BMI and different sleep parameters Bed time: 0.20 Night-time total sleep duration: −0.15	A later bed time was associated with a higher BMI in children, while shorter total sleep time at night correlated with increased BMI.
Martoni (2016) [32]	To quantify the difference in BMI based on varied sleep dimensions.		Mean (mins): 487.8			Beta-coefficient between sleep parameter and BMI Total sleep: −0.28 Sleep quality (Amplitude of circadian rhythm): −0.30	The findings indicated a significant negative association between total sleep time and BMI, suggesting that shorter sleep durations were linked to higher BMI values. Additionally, a lower amplitude of sleep rhythm was also associated with increased BMI.
Wang (2016) [33]	To identify the association between sleep duration and BMI in children	up to 5 years of age	≤10 h 11–12 h ≥13 h			OR (95% CI) OB: 1.70 (1.26–2.30) OW: 1.46 (1.24–1.71) OR (95% CI) OB: 1.00 OW: 1.00 OR (95% CI) OB: 1.09 (0.78–1.52) OW: 1.04 (0.88–1.23)	Children receiving less amount of sleep, (< 10 h) had a significantly elevated risk of being either obese or overweight than those receiving more than 10 h of sleep.
Alqaderi (2017) [34]	To identify the association between bed time and abdominal obesity in 10-year-old children, followed up after 2 years.	2 Years		Bed time: 10:00 p.m. Wake time: 6:00 am		Linear regression coefficient between bedtime and waist circumference−0.2 (0.07, 0.3)	A statistically significant association between late bed time and obesity as measured by waist circumference was reported.
Derks (2017) [35]	To investigate whether short sleep duration during infancy correlates with higher BMI during early childhood	5–6 years				Correlation coefficient (95% CI) between BMI and sleep duration at a varying time point during infancy 2 months: −0.024 (−0.033 to −0.015) 6 months: −0.021 (−0.031 to −0.010) 2 years: −0.051 (−0.074 to −0.027) 3 years: −0.043 (−0.066 to −0.019)	A statistically significant association between BMI and sleep duration at varying months of infancy is reported.
García-Hermoso (2017) [36]	To assess the link between childhood sleep patterns and negative weight status				Poor sleep quality (≥7)	OR for OB/OW (95% CI) 1.04 (0.94 to 1.34)	No significant link between the prevalence of obesity and overweight among children due to poor sleep quality was identified
Larsen (2017) [45]	To assess the gender-specific interaction between BMI and sleep duration					Bivariate correlation coefficient between sleep duration and BMI z Boys = −0.28 Girls = −0.15	Sleep duration in children was negatively associated with BMI z in boys, whereas no such association was observed in girls.
Del Pozo-Cruz (2017) [37]	To investigate relation between sleep duration and BMI in children		Sleep duration of different age groups (mins/day) 5–9 years: 637.31 10–14 years: 612.40			Regression coefficient between sleep duration and BMI (95% CI) −0.010 (−0.016 to −0.004) −0.016 (−0.023 to −0.010)	A significant negative association between sleep duration and BMI was reported, indicating that higher sleep duration results in lower BMI, and shorter sleep durations are prevalent in those with high BMI.
Rosi (2017) [38]	To assess the prevalence of obesity or overweight among children having varying sleep durations		Low Recommended High	Early bed/early rise Early bed/late rise Late bed/early rise Late bed/late rise		*p*-value between under-normal weight and OB/OW 0.008 *p*-value between under-normal weight and OB/OW 0.518	Sleep duration varies significantly among obese and overweight children. However, obesity/overweight and underweight/normal weight prevalence remain comparable across different sleep timings
Wang (2017) [39]	To identify any correlation between adiposity measures and sleep duration		Shortest Shorter Longer Longest			Liner regression coefficient between obesity measures and sleep durations BMI z-score: 0.00 (reference) Waist circumference: 0.00 (reference) Body fat%: 0.00 (reference) BMI z-score: −0.13 (−0.20, −0.06) Waist circumference: −1.26 (−1.91, −0.61) Body fat% %: 1.61 (0.77, 2.44) BMI z-score: −0.25 (−0.33, −0.18) Waist circumference: −2.12 (−2.80, −1.45) Body fat%: 1.24 (0.37, 2.12) BMI z-score: −0.11 (−0.23, −0.01) Waist circumference: −1.74 (−2.60, −0.88) Body fat% %: 0.17 (0.95, 1.30)	Children receiving more hours of sleep tend to have lower BMI z-scores and smaller waist circumferences compared to those with shorter sleep duration.
Taylor (2018) [40]	To identify the potential of sleep duration during infancy in predicting the changes in BMI in early childhood.	5 years				Regression coefficient (95% CI) between BMI z-score and sleep duration at varying time points during infancy 1 year: 0.00 (0.30) 2 years: 0.45 (0.40) 3.5 years: −1.57 (0.55) 5 years: −0.89 (0.54)	No significant correlation was observed between BMI z-score and sleep duration across several follow-up periods, except at the 3.5-year interval.
Derks (2019) [43]	To identify the association between sleep duration in infancy and change in BMI during childhood	14 years				OR (95% CI) between sleep duration at age 4 to BMI at the age of 14 years Low BMI: Ref Medium BMI: 1.07 (0.88 to 1.28) High BMI: 0.93 (0.70 to 1.25)	No significant association between sleep duration during mid-childhood and body composition at mid-adolescence was observed
Seo (2019) [44]	To identify the impact of varying sleep duration on the risk of obesity in adolescents					OR (95% CI) for OB/OW and sleep duration Long: 1.12 (0.49–1.02) Short: 1.18 (1.01–1.38) Very short: 1.76 (1.43–2.18) OR (95% CI) for OB and sleep duration Long: 1.28 (0.38–4.23) Short: 1.04 (0.82–1.32) Very short: 1.69 (1.25–2.29) OR (95% CI) for high waist circumference and sleep duration Long: 0.94 (0.28–3.09) Short: 1.25 (1.01–1.53) Very short: 1.49 (1.13–1.97)	Individuals with short and very short sleep duration were at higher risk of being overweight, obese, or having increased waist circumference compared to those who achieved optimal sleep.
Wang (2019) [41]	To identify the potential of sleep duration during infancy in predicting the changes in BMI in early childhood.	6 years				Linear regression coefficient for BMI z-score at 36 months and sleep duration at varying time periods 6 months: 0.001 14 months: −0.019	No significant association between BMI z-score at 36 months and sleep duration at either 6 or 14 months was reported
Zhang (2019) [42]	To assess the correlation between sleep duration and sleep quality with weight status in toddlers.					OR (95% CI) for OB Night sleep duration: 1.21 (0.62–2.37) Poor sleep quality: 1.00 (0.51–1.98)	No statistically significant risk of obesity was identified for nocturnal sleep duration and sleep quality

## Data Availability

No new data were created or analyzed in this study.
